# Prostasome-like vesicles stimulate acrosome reaction of pig spermatozoa

**DOI:** 10.1186/1477-7827-6-5

**Published:** 2008-01-30

**Authors:** Laura Siciliano, Vito Marcianò, Amalia Carpino

**Affiliations:** 1 Department of Cell Biology, Faculty of Pharmacy, University of Calabria, Cosenza, Italy; 2 Department of Experimental Medicine, Human Anatomy Section, University of Palermo, Italy

## Abstract

**Background:**

The presence of small membranous particles characterizes the male genital fluids of different mammalian species. The influence of semen vesicles, denominated prostasomes, on sperm functional properties has been well documented in humans, but their biological activity is scarcely known in other species. The present work investigated prostasome-like vesicles in pig semen for their ability to interact with spermatozoa and to affect acrosome reaction.

**Methods:**

Prostasome-like vesicles have been isolated from pig seminal plasma by high-speed centrifugation and Sephadex G-200 gel chromatography. Morphology of purified vesicles has been checked by scanning electron microscopy while their protein pattern has been investigated by SDS-PAGE. Then prostasome- like vesicles have been incubated with pig spermatozoa and their ability to interact with sperm has been tested by the aminopeptidase assay. In addition, the efficiency of vesicles to influence the acrosome reaction has been investigated by assessing the sperm acrosomal status by the PI/FITC-PNA (propidium iodide/fluorescein isothiocyanate-labeled peanut agglutinin) stainings.

**Results:**

Purified vesicles revealed a complex protein pattern with the occurrence of bands in the high, medium and low molecular weight range. However, the two major bands were observed at ~90 kDa and ~60 kDa. A vesicle-mediated transfer of aminopeptidase to sperm cells has been also detected. Furthermore, a significant increase of acrosome reaction extent has been revealed in spermatozoa incubated with prostasome-like vesicles in comparison to control sperm.

**Conclusion:**

This is the first report demonstrating that pig prostasome-like vesicles are able, in vitro, to interact with spermatozoa and to stimulate the acrosome reaction. These findings lead to hypothesize a transfer of molecules from vesicles to sperm membrane, thus sensitizing male gametes to undergo the acrosome reaction

## Background

Reproductive fluids of different mammals have revealed the presence of multilamellar vesicles, sized 50–500 nm, containing a lot of proteins, ions and a peculiar lipid composition with a high cholesterol/phospholipids ratio [1-3]. These membranous particles were firstly isolated from human semen and named prostasomes for their prostatic origin [4,5]. Human prostasomes play an important role in activation and protection of sperm cells for their different properties, such as sperm motility promotion, immunosoppressive and antibacterial capacities, antioxidant activity, etc [6-13]. Furthermore, a role of prostasomes in development of prostate cancer has been recently proposed [14].

The membrane vesicles isolated from non-human species were named prostasome-like vesicles for their different composition and amount with respect to human prostasomes. In addition, they showed multiple production sites, in fact, they were detected in semen but also in epididymal and/or seminal vesicle secretions of rat, bull and ram [15-19].

Recent studies identified prostasome-like vesicles in porcine semen revealing their morphology and biochemical composition [3,20]; however the biological properties of these vesicles have not been documented. The aim of this work was to investigate the functional role of pig prostasome-like vesicles assessing their *in vitro *ability to interact with sperm cells and to influence acrosome reaction.

## Methods

### Materials

*Chemicals *The reagents, all of reagent grade or better, were purchased from Sigma-Aldrich (Milan, Italy) unless otherwise indicated.

### Animals and sample collection

The investigation was conducted on semen samples from 16 fertile male pigs (*Sus scrofa domestica, Large White) *kept at "Swine Artificial Insemination Centre" (Rende, CS, Italy). The animals were 20 to 30 months-old and their weights were from 300 to 360 kg. Fresh ejaculates were collected by the gloved hand method and filtered immediately by Universal Semen bags (Minitub, Tiefenbech, Germany). Samples were then placed into an insulated container and brought to the laboratory within 1 h of collection.

### Isolation of prostasome-like vesicles

Prostasome-like vesicles were purified from individual pig semen samples as follows: ejaculates (10 mL) were centrifuged (800 g ×10 min) to harvest spermatozoa. The supernatant was diluted with 30 mM TRIS buffer, containing 130 mM NaCl, pH 7,6 and centrifuged at 13.000 g to eliminate residual spermatozoa and cell debris. The final supernatant was ultra-centrifuged at 105.000 g for 2 h (Beckman TL-100, Beckman Instruments Inc., Milan, Italy). The resulting pellet was resuspended in the same TRIS buffer and submitted to Sephadex G-200 gel chromatography to separate prostasomes from amorphous substance. The eluate was collected at 20°C at a flow rate of 6 mL cm^-2 ^h^-1. ^Vesicles not retained by the column were collected with the void volume. Fractions were examined for the activity of aminopeptidase, a marker of human prostasomes [21], then they were pooled and ultracentrifuged again at 105.000 g for 2 h to harvest the vesicles. Finally, the pellet was resuspended in a small volume of TRIS buffer and kept at -80°C until used.

### Scanning electron microscopy

Purification of prostasome-like vesicles was verified analysing their morphology by SEM. Purified vesicles were fixed with 2.5% glutaraldehyde in 0.1 M phosphate buffered saline, pH 7.3 and post-fixed with 1% osmium tetroxide in the same buffer. Then the samples were dehydrated in a graded series of acetone and by the critical point drying with liquid CO_2_ in an Emscope CPD 750 (Pabisch, Hitachi, Milan, Italy). Finally, the specimens were coated with Au by Polaron 5500 (Pabisch, Hitachi, Milan, Italy) and examined in a Jeol JSM 6031F scanning electron microscope (Jeol, Milan, Italy) at 30 kV.

### SDS-PAGE

Purified prostasome-like vesicles (30 μg protein) were diluted in Sample Buffer-Laemmli and processed by SDS-polyacrylamide gel electrophoresis (PAGE) on 11.5% acrylamide slab gels according to Laemmli (1970). The protein were visualized by Coomassie staining (20% ethanol, 5% acetic acid, and 0.07% Coomassie Brilliant Blue R250)(BioRad) Protein markers for molecular weight determination were purchased from Bio Rad.

### Sperm/vesicle interaction

Individual pig semen samples were centrifuged (800 g ×10 min) to harvest spermatozoa. Sperm cells were washed (6 min, 600 g) in Tyrode's capacitation medium (TALP: containing 2.0 mM calcium chloride, 2.7 mM potassium chloride, 0.5 mM magnesium chloride, 100.0 mM sodium chloride, 20.0 mM sodium bicarbonate, 0.4 mM sodium dihydrogen phosphate, 1.0 mM sodium pyruvate, 20.0 mM sodium lactate, 10.0 mM Hepes, and 0.6% BSA, 6.0 mM glucose, pH 7.4) [22].

After washing spermatozoa were resuspended in TALP and purified vesicles were added to reach with sperm a protein ratio of 2:1 [23]. Protein concentration was measured by the Bradford method [24]. The mixture was incubated at 39°C water bath and, at fixed intervals (0, 15, 30 and 60 min), aliquots of 50 × 10^6 ^spermatozoa were withdrawn, washed with TALP and assayed for sperm-vesicle interaction by the aminopeptidase assay. The experiments were performed in sperm cells from all the 16 pigs.

#### Aminopeptidase assay

Interaction of spermatozoa with prostasome like-vesicles was assessed by the aminopeptidase assay [25,26]. In fact, aminopeptidase is a prostasome marker which is normally absent from the sperm surface. Enzymatic activity was measured in semen vesicles, as well as in ejaculated spermatozoa before and after their incubation with vesicles. The evaluation of enzymatic activity was carried out according to Laurell [21]. Briefly, 5 mg of the [Suc(ala)3pNA] substrate were dissolved in 11 mL 0.2 M Tris-HCl buffer (pH 8). Samples were added to 1 mL of the substrate solution and, after 10 min, the 410 nm absorbance was read to detect the yellow hydrolysis product. Reaction specificity was verified using 0.1 M o-phenanthroline which caused the enzyme activity inhibition, an inactivation reversed by the subsequent addition of 0.033 M Zn(NO_3_)_2_.

### Acrosome reaction

Spermatozoa incubated with prostasome-like vesicles (Sv-sperm) and spermatozoa incubated only with buffer (Control sperm) were resuspended in TALP (5 × 10^6 ^sperm/mL), placed in a conical tube and cultured for 2 h in an atmosphere of 5% CO_2 _in air at 39°C. Then acrosomal status was monitored using the acrosome-specific fluorochrome fluorescein isothiocyanate-labeled peanut (*Arachis hypogaea*)agglutinin (FITC-PNA) in conjunction with DNA-specific fluorochrome propidium iodide (PI) as a viability test [27]. Briefly, sperm suspension (1 × 10^6 ^mL) was exposed to FITC-PNA (10 μg/mL) and propidium iodide (PI, 12 μmol/L) for 5 min at 39°C and then fixed by adding 1 μL of 12.5% (w/v) paraformaldehyde on 0.5 mol Tris/l (pH 7.4). The slides were immediately examined with an epifluorescence microscope (Olympus BX41) with a multiple fluorescence filter (U-DM-DA/FI/TX2) observing a minimum of 200 spermatozoa × slide (100× objective). Acrosomal status was assessed according to the staining patterns.

#### Staining patterns

Spermatozoa with a nuclear red PI staining were considered as dead cells while sperm cells without PI staining were considered as live cells.

Live sperm were classified in 2 main categories on the basis of the FITC-PNA staining as follows: i) acrosome-reacted cells with uniform green FITC-PNA fluorescence of acrosome cap ii) acrosome-intact cells without any fluorescence. Values were expressed as percentage. Three replicate experiments were performed for each semen sample.

### Statistical analysis

Results were expressed as percentage of spermatozoa in each class and the averages were compared by analysis of variance (one-way ANOVA) followed by the *t*-test at p < 0.05.

## Results

### Scanning electron microscopy

**Figure 1:**
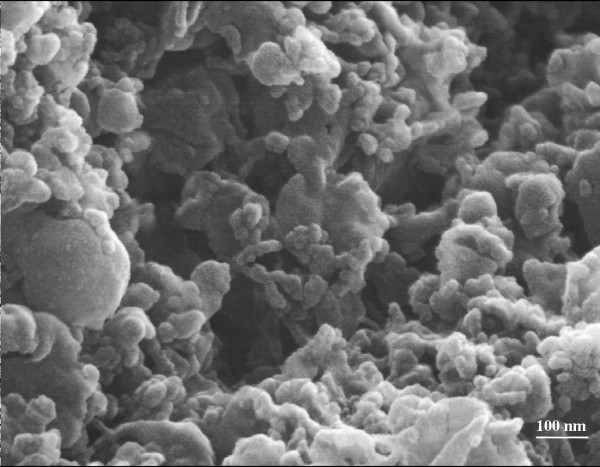
Representative scanning electron micrograph of purified vesicles from pig seminal plasma (× 80.000).

Purified vesicles were analysed by scanning electron microscopy to verify their morphology (Fig. 1). The analysis revealed the presence of membranous vesicles (Ø = 40–450 nm) morphologically similar to boar prostasome-like vesicles previously reported [3]

### SDS-PAGE

**Figure 2:**
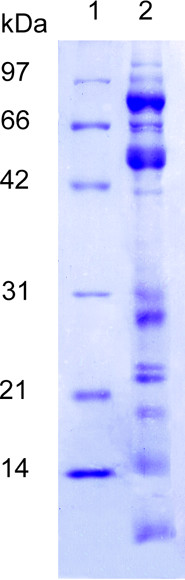
SDS-PAGE: Protein separation from 30 μg of pig purified prostasome-like vesicles on a Coomassie stained gel. *Lane *1: molecular weight standards *Lane *2: prostasome like-vesicles.

Following the *SDS-PAGE *protein separation, all the samples have revealed a complex protein pattern. Six distinct bands were observed in the high molecular weight range, with two major bands approximatively at 90 kDa and 60 kDa. In addition, some consistent bands were also detected in the medium and low molecular weight range (approximatively at 41, 31, 28, 25, 23, 19, 16 and 6 kDa). Figure 2 shows a Coomassie-stained SDS-PAGE gel of separated proteins from one representative sample

### Sperm-vesicle interaction

**Figure 3:**
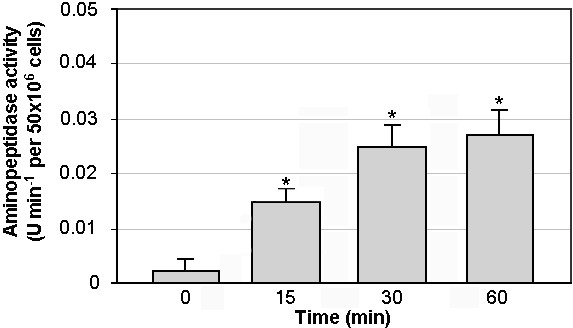
Aminopeptidase activity of pig spermatozoa before and after incubation with prostasome-like vesicles. At fixed intervals, aliquots of 50 × 10^6 ^cells were withdrawn and assayed for enzyme activity. Values are mean percentage ± SD. *Significantly different from sperm incubated at 0 time (p < 0.05).

Vesicles (2.5 mg protein) isolated from porcine semen revealed aminopeptidase activity : 0.175 U/min ± 0.06. Sperm samples revealed no aminopeptidase activity before the addition of vesicles. Spermatozoa incubated with vesicles displayed aminopeptidase activity which increased up to a 30 minute incubation (Fig 3). The 14 ± 4% enzyme activity was transferred from vesicles to the sperm cells (by 30 minutes).

### Acrosome reaction

**Figure 4:**
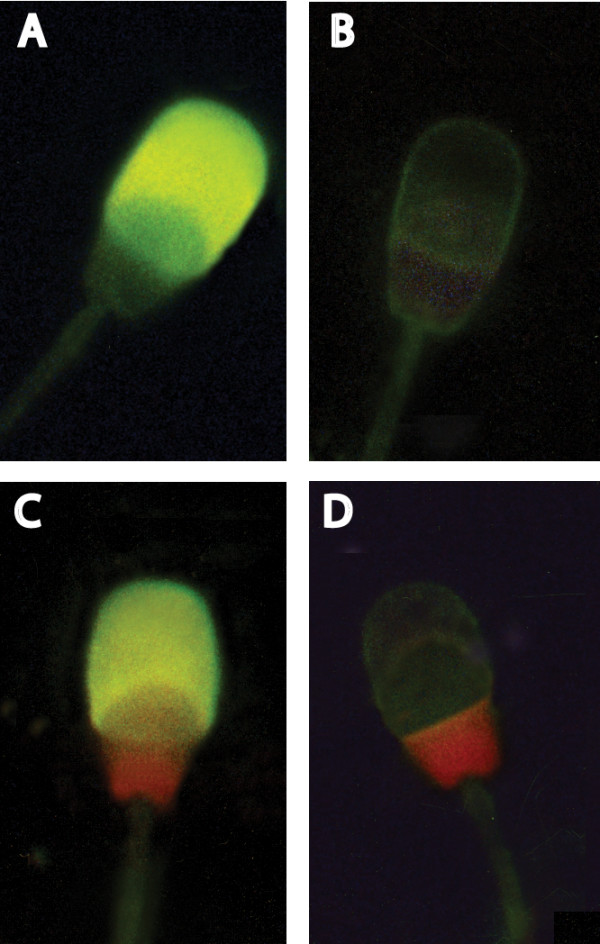
Fluorescence pattern of pig spermatozoa stained with FITC-PNA + PI for the assessment of acrosome status and sperm viability. Dead cells showing nuclear red PI fluorescence: C-D. Live cells without PI staining: A acrosome-reacted cells with uniform green FITC-PNA fluorescence of acrosome cap; B: acrosome-unreacted cells with no staining of acrosomal cap (original magnification ×1000).

Figure 4 shows a representative fluorescence pattern of pig spermatozoa stained with FITC-PNA + PI for the assessment of acrosome status and sperm viability

#### Sperm score

**Figure 5:**
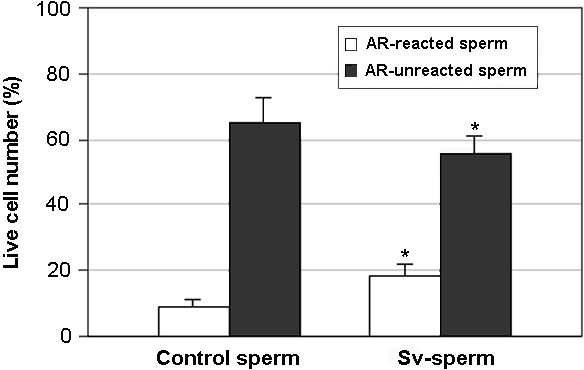
Effect of prostasome-like vesicles on acrosome status of pig sperm. Sperm were incubated with prostasome-like vesicles (sv-sperm) or with buffer (control sperm). *Black bars*: acrosome unreacted cells; *open bars*: acrosome reacted cells. Values are mean percentage ± SD. * Significantly different from control sperm (p < 0.05).

A similar incidence of dead sperm (PI positive cells) was observed in spermatozoa incubated with prostasome-like vesicles (16 ± 4%) and in control sperm (15 ± 4%). Furthermore, a higher percentage of acrosome- reacted cells (FITC-PNA positive cells) was detected in Sv-sperm (18 ± 4%) compared with the control sperm (9 ± 2%) (Fig. 5). Conversely, a lower incidence of acrosome-unreacted cells (no fluorescence) was detected in Sv-sperm in comparison with the control sperm (Fig. 5).

## Discussion

Submicron membranous vesicles have been detected in male genital tract fluids of several mammals such as rat, mouse, stallion, bull, ram and human [17,28]. Morphology, biochemical composition and origin of these vesicles have been investigated in different species but their physiological relevance in the reproductive process has been hypothesized only in humans. In fact, human prostasomes can interact with spermatozoa transferring them proteins, lipids and ions [12]. The capacity of prostasomes to interact with spermatozoa was firstly reported by Ronquist who hypothesized the occurrence of hydrophobic interactions [29]. However, later a pH dependent fusion (mainly at pH 5) between vesicles and sperm cells was demonstrated [23]. As a consequence of the fusion process, sperm membrane changes its composition and modifies the responsiveness to signals [28]. Therefore, the prostasome-sperm fusion can support the functional activity of sperm cells.

Recently, prostasome-like vesicles have been also detected in porcine semen. Particularly, they have been revealed in the sperm rich fraction of boar ejaculate, suggesting their origin from the combined contribution of epididymal and prostatic secretions [3]. Furthermore, boar prostasome-like vesicles share some characteristics with equine and human vesicles, such as the electron microscopy features, the high cholesterol/phospholipids ratio and the phospholipid composition [20].

The present paper has shown the SEM morphological features of pig semen vesicles together with their SDS-PAGE protein profile which was still unknown. Prostasome-like vesicles revealed a complex protein pattern with different bands in the high, medium and low MW range. The major bands were observed at ~90 kDa and ~60 kDa range, but some consistent bands were also detected in the 31 -6 kDa range.

This is the first report demonstrating the ability of pig prostasome-like vesicles to interact with spermatozoa. In fact, a vesicle-mediated transfer of aminopeptidase activity to spermatozoa has been detected after an appropriate incubation, as previously reported for equine vesicles [26]. A prostasome-mediated transfer of aminopeptidase activity to sperm cells was demonstrated also in humans [25] but it was the result of a fusion process, since it was carried out at pH 5.0. However, in humans, sperm deposition occurs in vagina, where physiological environment is acid, therefore, the experimental vesicle/spermatozoa fusion at pH 5.5 resembles the in vivo condition for the interaction.

For the uterine sperm deposition, vesicles of porcine semen meet spermatozoa before capacitation, following them in the female ducts. Therefore, it is reasonable to hypothesize their involvement in the last sperm maturation stages, addressed to the acquisition of the fertilizing capacity.

The present investigation has revealed that prostasome-like vesicles of the pig have the *in vitro *capacity to induce the sperm acrosome reaction, an exocytotic event necessary for the oocyte fertilization. Acrosome reaction follows the sperm capacitation, a complex process which modifies the plasma membrane composition, sensitizing sperm cells to physiological inducers of acrosome reaction.

The influence of sperm-vesicle fusion on acrosome reaction has been investigated only in humans, but with controversial results probably due to the complex events involved in this process. In fact, some authors suggested that prostasome-sperm fusion could delay the acrosome reaction [30], consistently with the cholesterol enrichment of sperm cells during the fusion process [2]. At the same time, other authors showed that prostasome-sperm fusion can stimulate the acrosome reaction making sperm cells more sensitive to the effect of progesterone [31]. This effect could be explain by the transfer of calcium and hydrolytic enzymes essential for acrosome reaction during the fusion process [32-34]. Our data showing a stimulating effect of porcine semen vesicles on the acrosome reaction are in agreement with Palmierini's report on human prostasomes [31]. Therefore, on the basis of our results, it is reasonable to hypothesize an *in vitro *vesicle-mediated transfer of substances to sperm cells, increasing membrane responsiveness to undergo the acrosome reaction. To date, only lipid composition has been extensively investigated in semen vesicles of the pig. Future studies on vesicle composition, and particularly their proteomic analysis, will aid to clarify the specific components involved in the sperm-vesicle interaction and in the acrosome reaction induction.

## Conclusion

Membrane vesicles of male genital fluids can be considered the new insight in the post-testicular maturation of mammalian spermatozoa. This is the first report demonstrating that prostasome-like vesicles from pig semen are able, *in vitro*, to interact with spermatozoa and to stimulate the acrosome reaction. These findings lead to hypothesize a transfer of molecules from vesicles to sperm membrane, thus sensitizing male gametes to undergo the acrosome reaction

## Authors' contributions

LS the author responsible for performing the experiments and participating in the analysis and interpretation of data.

VM the author responsible for electron microscopy analysis

AC the author responsible for conception, design, analysis and interpretation of data as well as of drafting manuscript
